# Soleus Muscle H-reflex: Reference Data of Adult Population From a Tertiary Care Center in India

**DOI:** 10.7759/cureus.59083

**Published:** 2024-04-26

**Authors:** Binay K Pandey, Ashok Kumar, Abhay Ranjan, Josni Pandey

**Affiliations:** 1 Neurology, Indira Gandhi Institute of Medical Sciences (IGIMS) Patna, Patna, IND; 2 Physiotherapy, Indian Institute of Health Education and Research, Patna, IND

**Keywords:** h-reflex, hoffmann reflex, soleus h-reflex, reference values, nerve conduction studies

## Abstract

Introduction

The Hoffmann reflex (H reflex) is one of the most studied reflexes in human neurophysiological literature. Detection of the H reflex is useful in the diagnosis of early polyneuropathy, S1 radiculopathy, early GBS, tibial neuropathy and sciatica, and sacral plexopathy. The H reflex is also used as a tool to measure the excitability of the nervous components of the arc, regardless of the sensitivity of the sensory organs. The monosynaptic nature of reflex circuits makes H-reflex an attractive tool for clinical neurophysiology and research.

Objective

The objective is to create reference data of soleus H-reflex latency in an adult population from a tertiary care center in India.

Materials and methods

Seven hundred eighty-four healthy volunteers underwent a physical examination and brief electrophysiological examination before elicitation of the soleus H reflex of both lower extremities using standard techniques. Reference values ​​are expressed as mean ± standard deviation as well as the third and 97th percentiles for latency as the dependent variable.

Results

The study population included 346 (44.1%) women and 438 (55.9%) men. The men were aged 40.46 ± 14.76 years, and the women were aged 41.63 ± 13.49 years. The average weight of the men was 73.32 ± 10.28 kilograms, and the women were 62.91 ± 7.46 kilograms. The average height of the men was 172.06 ± 4.22 cm, and the women were 159.12 ± 2.42 cm.

The third and 97th percentiles for H-reflex latency on the right side were 22.86 ms to 34.22 ms and on the left side were 22.86 ms to 35.39 ms. The average right tibial H latency and left tibial H latency were 28.18 ± 2.59 ms and 28.14 ± 2.70 ms, respectively.

Conclusion

A sizable subject population was used to provide reference data for this study. Because of the huge sample size and nearly appropriate coverage of different age groups, reference ranges have been established for various age, height, and BMI groups.

## Introduction

The Hoffmann reflex (H reflex) is an electrically induced reflex that corresponds to the mechanically induced spinal stretch reflex, first described by Paul Hoffmann in 1910. The main difference between the H-reflex and the spinal stretch reflex is that the H-reflex bypasses muscle spindles. It is a valuable tool for assessing the regulation of monosynaptic reflex activity in the spinal cord. The H-reflex is an estimate of alpha motor neuron (αMN) excitability when alpha motor neuron (αMN) presynaptic inhibition and intrinsic excitability remain constant [1,2]. This measurement can be used to assess the response of the nervous system to various neurological diseases, musculoskeletal injuries, treatment use, pain, physical training, and performance of motor tasks [2].

Indira Gandhi Institute of Medical Sciences (IGIMS), Patna, is an autonomous tertiary care institute. Due to the lack of population-specific reference values, the institute relies on data from textbooks or the literature. So, there was a need to generate its' own reference data.

Many of the previous studies have methodological limitations like inadequate sample size, biased sampling, and used conventional statistical analysis, etc. Normative Data Task Force (NDTF) formed by the American Association of Neuromuscular & Electrodiagnostic Medicine (AANEM) developed a set of quality criteria to define the important methodological issues required of studies that investigate nerve conduction normative values [3]. These criteria can serve as benchmarks for future normative studies. The present study has taken into consideration these criteria to generate reference data.

## Materials and methods

After obtaining approval from the Institutional Ethics Committee (1614/IEC/IGIMS/2020), healthy volunteers, who gave their written consent, were evaluated and included in the study if they met the pertinent inclusion criteria. The study was conducted between June 2020 and February 2023. Participants were recruited from the inhabitants of the IGIMS residential complex as well as the community dwellers apart from the persons accompanying the patients.

All the participants underwent a detailed comprehensive neurological examination [4]. A brief electrophysiological evaluation was also done for each participant using the standard techniques to rule out asymptomatic polyneuropathy [5].

The Neurological examination included manual muscle testing, deep tendon reflexes, sensation for vibration, pinprick, temperature, light touch and proprioception, gait, and tandem gait. Physical examination result was considered abnormal if there was any degree of weakness, decreased or absent reflexes, decreased or absent sensation for any sensory modality, and any gait abnormality. For individuals with no symptoms of peripheral neuropathy, a normal clinical neurological examination, and normal electrophysiological evaluation were included.

Individuals with a history of lumbosacral radiculopathy, diabetes mellitus, hypothyroid, any neurological or neuromuscular transmission disorder, history of alcoholism, smoking, or taking any drug known to cause neuropathy were excluded.

Height and weight were measured using a stadiometer and digital weighing machine respectively. Body Mass Index (BMI) was calculated by dividing weight in kilograms by height in meter square. Age to the nearest year was recorded. Skin temperature was recorded bilaterally at the mid-popliteal crease using a digital dual-input K-type thermometer. The limb temperature was kept at 33 to 34 degrees Celsius throughout the evaluation [6].

Recording technique

Preparation of the skin was done to reduce the skin impedance and if required, the skin was shaved before fixing the electrodes.

Position of the subjects

Soleus H-reflex latency was recorded with participants in a prone lying position in a quiet room on a comfortable examination table. The head was maintained in mid position to control the possible effect of asymmetrical tonic reflex. The knee was slightly flexed to about 20 degrees by placing a pillow under the leg, and the ankle was freely positioned in the plantarflexed position to relax the gastrocnemius to help reduce any depressive influence on H-reflex.

Position of the electrodes

**Figure 1:**
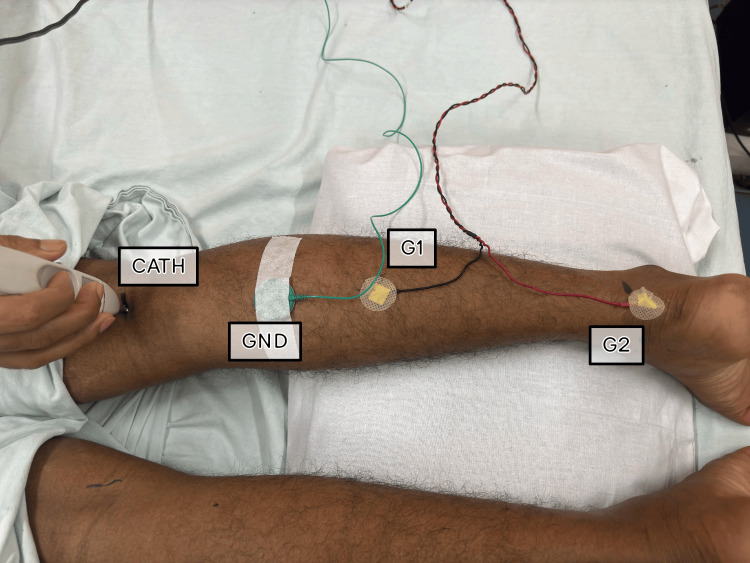
Position of the Subject, and Position of the electrodes G1: Active Electrode, G2: Reference Electrode, GND: Ground Electrode, CATH: Cathode

For eliciting Soleus H-reflex, a Nicolet Viking Quest machine was used. While eliciting Soleus H- reflex subjects were placed prone. The knee was passively flexed to locate and mark the popliteal crease. The lower leg was then lowered onto a pillow with the foot hanging over the edge of the table for optimal relaxation. A second mark was placed on the posterior calcaneus. The distance between these points was measured, and the active electrode (G1) was placed midway between the marks. The reference electrode (G2) was placed over the posterior calcaneus. The ground electrode (GND) was placed proximally to the active electrode (Figure 1).

Stimulation

**Figure 2:**
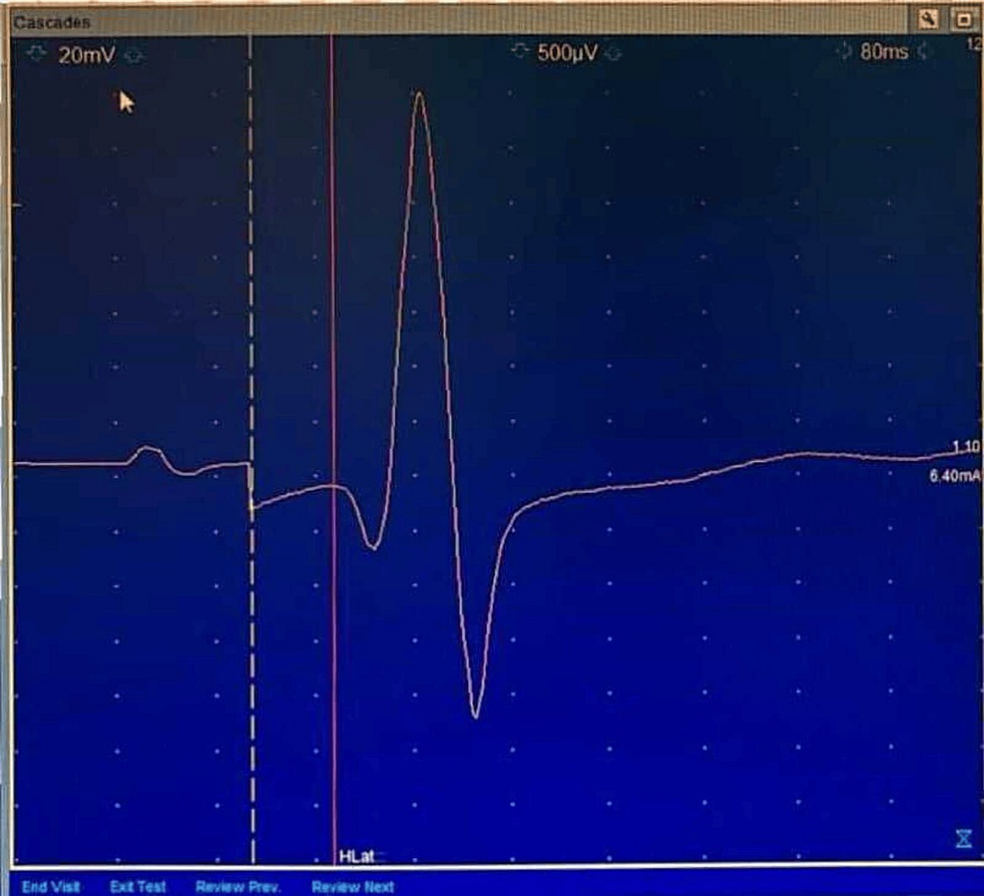
A typical H-Reflex

Submaximal stimuli were applied at the mid-popliteal crease at a stimulus duration of 1 ms, with the cathode proximal to avoid the anodal block. Stimuli were applied at approximately five-second intervals at random to avoid habituation errors. Sensitivity was kept at 500 microvolt/division, low-frequency filter at 2-3 Hz, High-frequency filter at 10 kHz, and sweep at 10 millisecond/division. Stimulus intensity was adjusted to obtain the maximal amplitude, and the latency to the first deflection from the baseline. Soleus H-reflex latency was measured from the beginning of the 1 ms stimulus to the take-off of the H wave from baseline (Figure 2).

Statistical analysis

Statistical analysis was done using IBM SPSS Statistics for Windows, Version 26.0 (IBM Corp., Armonk, NY). Continuous variables were reported as mean and standard deviation as well as in different quantiles.

## Results

The study participants included 346 (44.1%) women and 438 (55.9%) men between the age group of 18 to 65 years. The mean age of men was 40.46 ± 14.76 years, and that of women was 41.63 ± 13.49 years. The average weight of the men was 73.32 ± 10.28 kg, and the women were 62.91 ± 7.46 kg. The average height of the men was 172.06 ± 4.22 cm, and the women were 159.12 ± 2.42 cm (Table 1). Participants were divided into various groups as per their age, height, body mass index, and gender (Table 2).

**Table 1:** Basic data of the participants Descriptive statistics about basic data of participants presented as mean ± SD

Basic Data	Male (N=438)	Female (N=346)	Total (N= 784)
	Mean ± SD	Mean ± SD	Mean ± SD
Age (years)	40.46 ± 14.76	41.63 ± 13.49	40.97 ± 14.22
Height (cm)	172.06 ± 4.22	159.12 ± 2.42	166.35 ± 7.34
Body Weight (kg)	73.32 ± 10.28	62.91 ± 7.46	68.73 ± 10.50
BMI (kg/m^2^)	24.74 ± 3.21	24.84 ± 2.85	24.78 ± 3.06

**Table 2:** Different age, gender, BMI, and height groups Study participants were divided into age, height, and BMI categories

Characteristics	Group	Parameters
Age (years)	Group 1. Young Adults	18 to 35
	Group 2. Middle Aged	36 to 55
	Group 3. Older Adults	56 and above
Gender	Group 0	Male
	Group 1	Female
BMI (kg/m^2^)	Group 1. Underweight	Less than 18.5
	Group 2. Healthy weight	18.5 to 24.9
	Group 3. Overweight	25 to 29.9
	Group 4. Obese	More than 30
Height (cm)	Group 1	Less than 160
	Group 2	160 to 169
	Group 3	170 to 179
	Group 4	Equal to and more than 180

The average right Soleus H-reflex latency and left Soleus H-reflex latency were 28.18 ± 2.59 ms and 28.14 ± 2.70 ms, respectively. The third and 97th percentiles for Soleus H- reflex latency on the right side was 22.86 ms to 34.22 ms and on the left side was 22.86 ms to 35.39 ms. Detailed reference data for various age, height, and BMI groups are presented in Tables 3-6.

**Table 3:** Soleus H-reflex latency (ms) for age groups The table shows data on soleus H-reflex latency (ms) by age group, including participant count (N), mean ± SD, and percentiles (third to 97th) for both sides.

Age Groups	N	Mean ± SD		Percentiles (3rd – 97th)	
		Right	Left	Right	Left
Group 1. Young Adults	305	27.46 ± 2.19	27.36 ± 2.42	22.86-30.80	22.86-31.88
Group 2. Middle Aged	327	28.38 ± 2.75	28.29 ± 2.84	23.39-35.10	23.54-35.39
Group 3. Older Adults	152	29.23 ± 2.45	29.36 ± 2.41	24.79-34.22	25.35-33.59
Total	784	28.16 ± 2.61	28.14 ± 2.70	NA	NA

**Table 4:** Soleus H-reflex latency (ms) for height groups The table shows the number of participants (N), mean soleus H-reflex latency with standard deviation (Mean ± SD), and percentile ranges (third to 97th) for both right and left sides in each height group.

Height Groups (cm)	N	Mean ± SD		Percentiles (3rd – 97th)	
		Right	Left	Right	Left
Group 1. < 160	339	27.81 ± 2.60	27.73 ± 2.83	23.33-32.55	22.60-32.86
Group 2. 160-169	64	28.23 ± 2.39	28.46 ± 2.44	24.27-33.44	23.80-33.39
Group 3. 170-179	373	28.44 ± 2.61	28.42 ± 2.56	24.79-35.00	24.53-34.79
Group 4. ≥ 180	8	29.51 ± 2.60	29.49 ± 3.46	25.47-33.44	23.85-34.79
Total	784	28.16 ± 2.60	28.13 ± 2.70	NA	NA

**Table 5:** Soleus H-reflex latency (ms) for BMI groups The table shows the number of participants (N), mean soleus H-reflex latency (Mean ± SD), and percentile ranges (third to 97th) for both right and left sides within each BMI group.

BMI Groups (kg/m^2^)	N	Mean ± SD		Percentiles (3rd – 97th)	
		Right	Left	Right	Left
Group 1. Underweight	36	26.92 ± 2.33	26.98 ± 2.23	20.89-29.84	23.28-30.10
Group 2. Healthy	331	27.87 ± 2.47	27.87 ± 2.58	23.44-33.44	23.49-34.79
Group 3. Overweight	390	28.47 ± 2.68	28.42 ± 2.79	23.39-34.48	22.86-34.79
Group 4. Obese	27	28.78 ± 2.50	28.83 ± 2.67	23.39-34.58	23.85-37.10
Total	784	28.16 ± 2.60	28.14 ± 2.71	NA	NA

**Table 6:** Soleus H-reflex latency (ms) for gender The table shows the number of participants (N), mean soleus H-reflex latency (Mean ± SD), and percentile ranges (third to 97th) for both right and left sides within each gender group.

Gender	N	Mean ± SD		Percentiles (3rd – 97th)	
		Right	Left	Right	Left
Female	346	27.88 ± 2.45	27.83 ± 2.69	23.39-32.50	22.86-32.86
Male	438	28.38 ± 2.70	28.38 ± 2.70	23.75-34.22	23.54-34.79
Total	784	28.16 ± 2.60	28.14 ± 2.71	NA	NA

Table 3 summarizes the soleus H-reflex latency measures for three age groups: young adults (Group 1), middle-aged (Group 2), and older adults (Group 3). The mean H-reflex latency values varied from 27.46 ms to 29.23 ms across age groups, with standard deviations ranging from 2.19 ms to 2.75 ms.

In terms of percentiles, the third to 97th percentile ranges varied by age group on both the right and left sides. For example, in Group 1 (Young Adults), the third to 97th percentile range for the right side was 22.86 ms to 30.80 ms, whereas the left side ranged from 22.86 to 31.88 ms. Similarly, in Group 3 (Older Adults), the comparable ranges were 24.79 ms to 34.22 ms on the right side and 25.35 ms to 33.59 ms on the left.

Overall, the study shows that soleus H-reflex latency increases with age, with older persons having higher latency than young and middle-aged adults. However, there is variation within each age group, as evidenced by the standard deviation and percentile distributions.

Each group represents a variety of heights, allowing for comparisons between height groups. N (Number of Participants) refers to the sample size for each height category. The Mean ± SD (Standard Deviation) represents the average soleus H-reflex latency for both the right and left sides, as well as the standard deviation for each height group. Percentiles (third - 97th) shows the range in which a specific percentage of the data falls for each height group.

The mean soleus H-reflex latency tends to rise significantly with increasing height groups, as seen by the trend from Group 1 to Group 4. Standard deviations within each height group provide information on the variability of H-reflex latency data. The standard deviations are generally constant across height categories, indicating similar degrees of variability within each group.

Group 4 (≥ 180 cm) has the greatest mean H-reflex latency values, indicating that taller persons may have slightly longer latencies than shorter individuals. Group 4 (≥ 180 cm) has a limited sample size (N = 8), potentially affecting the credibility of the standard deviation estimate.

Overall, the data show that as height increases, the mean soleus H-reflex latency values tend to increase significantly. For example, the average H-reflex latency rises from 27.81 ms in the “< 160 cm” group to 29.51 ms in the “≥ 180 cm” group. The “≥ 180 cm” group has a small sample size (N = 8), which may limit the findings' generalizability.

These data imply a possible link between height and soleus H-reflex latency, with taller people having slightly longer H-reflex latencies. The data show that as BMI increases from underweight to obese, the mean soleus H-reflex latency values rise. For example, the average H-reflex latency rises from 26.92 ms in the underweight group to 28.78 ms in the obese group. However, it is worth noting that the sample size for the obese group is quite small (N = 27), which may limit the generalizability of the findings.

The standard deviations within each BMI category indicate variation in H-reflex latency measures. The percentile ranges show the distribution of H-reflex latency measurements within each BMI group.

These data point to a possible link between BMI and soleus H-reflex latency, with those with higher BMIs having slightly longer H-reflex latencies. However, additional study with bigger sample sizes in each BMI group is needed to corroborate these findings.

Table 6 shows N (Number of Participants), which indicates the sample size for each gender category. The study included 346 female and 438 male participants.

Mean ± SD (Standard Deviation): Indicates the average soleus H-reflex latency for both the right and left sides, including the standard deviation for each gender group. Percentiles (third to 97th) indicates the range within which a specific percentage of the data falls for each gender group.

According to the data, males have a somewhat larger mean soleus H-reflex latency than females. Standard deviations within each gender group provide information on the variability of H-reflex latency measures. The variability appears to be slightly higher in males than females, particularly on the left side.

Percentile ranges depict the distribution of H-reflex latency values within each gender group. Both genders had similar percentile ranges, implying that the distribution of soleus H-reflex latency values is similar between males and females. Furthermore, percentile ranges show the distribution of H-reflex latency data among each gender group. For example, for females, the third to 97th percentile range on the right side is 23.39 ms to 32.50 ms, whereas for men it is 23.75 ms to 34.22 ms.

These data point to potential gender differences in soleus H-reflex latency, with males having somewhat longer latencies than females. In summary, this perspective sheds light on how soleus H-reflex latency varies across genders.

Overall, the study looked at soleus H-reflex latency (ms) in different demographic categories, including age, height, BMI, and gender. The data highlight a number of notable patterns given as follows.

Age groups

As people get older, their soleus H-reflex latency tends to increase. Older persons had longer H-reflex latencies than young and middle-aged adults. However, heterogeneity within each age group indicates that neuromuscular function varies by individual.

Height groups

Taller individuals have slightly greater mean soleus H-reflex latency values than shorter individuals. However, the sample size for the tallest height group is small, necessitating caution in interpretation.

BMI groups

As BMI grows from underweight to obese, so does soleus H-reflex latency. This shows a possible link between higher BMI and longer H-reflex latencies, but more study with bigger sample numbers is required to corroborate these findings.

Gender

Males often have slightly greater mean soleus H-reflex latency values than females. The gender difference in H-reflex latency may represent physiological differences between males and females, but more research is needed to investigate other contributing causes.

## Discussion

Many authors have studied the normal ranges of Soleus H-reflex as presented in Table 7, but there are methodological limitations such as insufficient sample size, sampling bias, and use of conventional statistical analysis, etc. [7-11].

**Table 7:** Soleus H-reflex latency reference values (ms) of this study in comparison with another studies This table compares the Soleus H-reflex latency values (Mean ± SD or Range) found in the current study to those from other studies, offering insight into the consistency or fluctuation in these values across different populations or methodology.

Study	N	Soleus H-reflex latency
Present Study	784	28.2 ± 2.6
Khosrawi et al. [7]	75	27.94 ± 1.6
Palve et al. [8]	150	24.3‑32.0
Huang et al. [9]	101	28.4 ± 1.6
Kimura [10]	NA	29.5 ± 2.4
Buschbacher [11]	251	30.3 ± 2.4

Large sample size increases the power and precision of reference data studies as well as reduces the mean square errors (MSEs) [3]. It has been found out that with a sample size of 100, the MSEs decrease by 80% [12]. Some authors have suggested that a sample size of around 300 is optimal [13]. This study has presented reference data using a very large subject population (n=784).

As per the NDTF criteria, apart from recruiting community-dwelling healthy subjects, a detailed comprehensive neurological examination and a brief electrophysiological evaluation of the participants were also done as a criterion for the normalcy of participants [3].

Age, Height, and BMI are those variables that may influence nerve conduction parameters [14]. The NDTF suggests a wide distribution of participants’ age and subset of age to be adequately represented. In this study, we could create separate reference ranges for various age, height, and BMI groups due to the large sample size and near-adequate representation of various age groups. This study has presented reference data using a very large subject population (n=784).

The mean Soleus H-reflex latency revealed in the current study (28.2 ms) is somewhat compatible with the findings of other investigations, such as Khosrawi et al. (27.94 ms), Huang et al. (28.4 ms), and Kimura (29.5 ms). This shows that these investigations agree on the average latency of the Soleus H-reflex.

However, mean latency values vary between investigations, with Palve et al. finding a larger range (24.3 to 32.0 ms) and Buschbacher reporting a somewhat greater mean latency (30.3 ms). This variation could be attributed to differences in sample characteristics, measuring methodology, or other variables.

The current study has a significantly bigger sample size (N=784) than several of the other studies cited. A greater sample size typically improves the reliability and generalizability of findings, implying that the reported mean values as well as the percentile values in the current study may be more representative of the community.

The limitation of this study is the issue of limited sample size in some subgroups. In the BMI group, the obese subgroup has a relatively small sample size (N = 27) which may affect the generality of the findings. Similarly, height subgroup 4 (≥ 180 cm) has a limited sample size (N=8) potentially affecting the credibility of the standard deviation estimate. Further research with larger sample sizes in each BMI and height categories is warranted to further confirm these observations.

## Conclusions

In summary, this study followed the NDTF criteria and used a sizable healthy subject population to provide reference data for Soleus H-reflex latency. Because of the huge sample size and nearly appropriate coverage of different age groups, reference ranges have been established for various age, height, and BMI groups.

This study of soleus H-reflex latency across diverse demographic groups sheds light on the factors that influence the reference data for soleus H-reflex latencies. The observed trends indicate that age, height, BMI, and gender may all influence H-reflex latency.

The outcome of the study showed that increasing age is associated with longer soleus H-reflex latencies, with older adults transmitting neural information at a slower rate than younger people. This age-related variance emphasizes the necessity of taking into account age-related variations when measuring and interpreting H-reflex measures.

BMI appears as a potential predictor of soleus H-reflex latency, with larger BMI categories corresponding to longer latency values. More research is needed to validate the relationship and investigate the underlying mechanisms. Furthermore, gender differences in soleus H-reflex latency are noted, with males typically having somewhat longer latencies than females.
